# Supplementary material for: Chromothripsis during telomere crisis is independent of NHEJ, and consistent with a replicative origin
Source: Genome Res. 2019 May;29(5):737–49. doi: 10.1101/gr.240705.118 (PMC6499312; doi:10.1101/gr.240705.118)
Supplement: Supplemental Material [file supp_gr.240705.118_Supplemental_file_1.zip › contigs/annotated_contigs/DB78/contig.2.DB78_length_703_mean_cov_12.1564722617.docx]

**DB78_length_703_mean_cov_12.1564722617**

```
CCATAAAATACACCAAATATATGCACTATAGTTTATTTAACCTGTTCCCGAGTGTCTGTGAGTATATCACTGTATTTGATTTTTCATGT
 >chr6:159109157-159109534 + E=5e-215
TATAGATAACTCTGTGATGAACGTCTTGTACAGGTATTTTGACATGTCTGTTTATTTTTGGGGGGTAAATTCTTAGAAGAATGATTGGT

CAAGTGTATGAATACTTGAAAGGCTTTTGATGTTTTGCCAAATTACCCTCTGGGAGTGTGTGCTTATTTACAACAGGCTGGCAGGGTAC

ACAAATTTCCTTCTTCCCACACTCTGGCCCCCATTACATTCTGAGCTTTGAAGCATGATTTCAAATTACATTCACGTAAAAAACTAGAC

GCCAGGCTCAGTGGCTCATGC|TAACTCTTTACAATCATCAGGAGGCCCTATCTAATTTTTTTTCCTCCCAATCCTTGATGTTCCTGGT
                       >chr6:159110979-159111305 + E=2e-184
ACCATTTGTTACATAACCATTCCCTTTCCCATTGCGTTGTAACCCAGGGCCACTGATTGGCACAGCGTGGGGCACCACTCTCCTTGTGC

CCAGCCCTGCGCTCCAGCGGGTGCTGTCTCATAGAGGACACACGGTGCCCTCTCCTTCACCTGCAACTCTTGGCAATTTGGCCCTAGCC

AGATTTTGAATGTTTGAGAGCAGGAGCTATGAGTAAAGGAATAAGTGAATAAATAAATTGAGCAGGGATCTGCTCATTTGG
```
